# Color of Intra-Ocular Lens and Cataract Type Are Prognostic Determinants of Health Indices After Visual and Photoreceptive Restoration by Surgery

**DOI:** 10.1089/rej.2014.1613

**Published:** 2015-04-01

**Authors:** Masahiko Ayaki, Kazuno Negishi, Yoshimi Suzukamo, Kazuo Tsubota

**Affiliations:** ^1^ Department of Ophthalmology, Keio University School of Medicine, Tokyo, Japan.; ^2^ Department of Physical Medicine and Rehabilitation, Tohoku University Graduate School of Medicine, Sendai, Japan.

## Abstract

***Background:*** This study compared post-operative quality of life and sleep according to the type of cataract opacity and color of the implanted intra-ocular lens (IOL).

***Methods:*** This is a cohort study and participants were 206 patients (average age 74.1 years) undergoing cataract surgery with the implantation of a clear ultra-violet (UV)-blocking IOL (C) or a yellow blue-light-blocking IOL (Y). Participants were evaluated using the National Eye Institute Visual Function Questionnaire (VFQ-25) and Pittsburgh Sleep Quality Index (PSQI) before surgery and 2 and 7 months after surgery. Changes in sub-scale scores of VFQ-25 and PSQI were compared.

***Results:*** Sub-scale analyses for improvement after surgery revealed significant differences in ocular pain scores on the VFQ-25 (Y>C; the higher the score, the better the outcome). Furthermore, there were significant differences between the two IOLs in terms of the sleep latency score (C>Y) and sleep disturbances score (C>Y). A posterior sub-capsular cataract was significantly correlated with improvements in ocular pain and sleep latency scores. These effects were successfully represented by the change in scores rather than absolute post-operative scores because individual standard of response may often change after intervention, recognized as a response shift phenomenon in patient-reported outcome study. Regarding seasonal differences, patients who had surgery in summer exhibited relatively better sleep quality than those who had surgery in winter.

***Conclusions:*** Analysis of sub-scales of health indices demonstrated characteristic prognoses for each IOL and cataract type. Cataract surgery may potentially contribute to systemic health in older adults.

## Introduction

Quality of life (QOL) is an important indicator of surgical outcome, in addition to survival, physical function, mental status, and/or laboratory results. One of the issues in measuring QOL in cataract patients may be a “response shift,”^1,2^ because cataract surgery results in considerable improvement that may occasionally evoke a patient's recognition of their own visual disability for the first time in the days following surgery. Several reports have documented the beneficial effects of cataract surgery on health indices in older adults, specifically life span, falls, mobility, sleep quality, and cognitive function.^3–6^

The color of an intra-ocular lens (IOL) is an emerging topic of interest among cataract surgeons, particularly the question as to whether a blue-light filter has any beneficial effects in terms of visual function, age-related macular degeneration, and circadian rhythm.^7–10^ Theoretically, a blue-light filter should effectively reduce phototoxicity and glare originating from blue light, as well as photophobia characterized by an excessive sensitivity to light that causes ocular pain and headache.^11,12^ However, blue-light filters are yellow-tinted in appearance (yellow being the complementary color), and this may have adverse effects on color and scotopic vision, as well as disrupting circadian photoentrainment.^13,14^ There is one large comparative study that has investigated this issue, and, in that study, overall sleep quality and sleep latency improved after removal of cataract regardless of the type of IOL implanted.^15^ The authors of that study concluded that implantation of a blue-light filter IOL did not have a negative impact on the sleep–wake cycle. In ophthalmologic practice, cataract surgeons can now select any one of three types of IOL, namely those that block UV, violet, or blue light. Previous studies reported that both UV- and blue-light-blocking IOLs had beneficial effects on systemic health in terms of vision-related QOL, sleep quality, and gait speed.^16–18^

Light is essential not only for vision but also for systemic health to control homeostatic mechanisms, including circadian rhythm, sleep, mood, metabolism, and the endocrine system.^19^ Light therapy itself is an established treatment for seasonal affective disorder caused by decreased exposure to daylight^20,21^; therefore, cataract surgery could be thought of as a sort of light therapy by providing better irradiance to the eye following the removal of a dense light filter. ^22^ The amount of light reaching the retina is determined by the area of the pupil, the optical density of the lens, and the optical density of the macular pigment.^23^ The spectral specificity of the lens is largely confined to short wavelengths, and this may have a major impact on photoreception in cataract patients.^13,14^

The aim of the present study was to evaluate cataract surgery as a treatment strategy to improve systemic health as a result of improvements in light transmittance and vision. We used two validated questionnaires to evaluate QOL, namely the National Eye Institute Visual Function Questionnaire (VFQ-25; Japanese version 1.4)^24^ and the Japanese version of the Pittsburgh Sleep Quality Index (PSQI; normal range<6).^25^ Both questionnaires were self-administered. The sub-scales on the PSQI are subjective sleep quality, sleep latency, sleep duration, habitual sleep efficacy, sleep disturbances, sleep medication, and daytime dysfunction. The sub-scales on the VFQ-25 are general health, role limitations, dependency, social function, mental health, driving, ocular pain, general vision, near vision, distance vision, peripheral vision, and color vision.

## Methods

The present study was approved by the local institutional review boards of Mita Hospital, International University of Health and Welfare, and Saitama National Hospital. All patients provided written informed consent prior to participating in the study. The inclusion criterion was the presence of lens opacity accounting for patient's visual symptoms, and we observed consecutive cataract patients indicated for surgery. The time frame of surgery was May, 2011, to March, 2013, with the follow-up assessment running from January, 2012, to October, 2013. The exclusion criterion was major intra- or post-operative complications. In all, 206 consecutive patients undergoing unilateral or bilateral cataract surgery were enrolled in the study and all of them agreed to participate in the study. Table 1 details patient demographics in both IOL groups. There were significant differences in pre-operative visual acuity in the better eye and the presence of nuclear sclerosis between patient groups implanted with a clear versus yellow IOL (Table 2).

**Table 1.** Patient Demographics and Health-Related Characteristics

	*Clear IOL*	*Yellow IOL*	p *value*
No. patients	71	135	
No. men/women	30/41	53/82	NS^a^
Age (years)	74.5±7.7	73.9±9.2	NS^b^
Height (cm)	157±9	154±9	NS^b^
Weight (kg)	55±13	56±11	NS^b^
BMI (kg/m^2^)	21.9±4.6	23.5±3.5	NS^b^
SBP (mmHg)	133±20	130±17	<0.05^b^
% Diabetic (HbA1c>5.8%, JDS)	30.6	26.1	NS^a^

Unless indicated otherwise, data are given as the mean±standard deviation (SD).

^a^ Chi-squared test.

^b^ Unpaired *t*-test.

IOL, intra-ocular lens; NS, not significant; BMI, body mass index; SBP, systolic blood pressure; HbA1c, glycated hemoglobin; JDS, Japan Diabetes Society.

**Table 2.** Results of Ophthalmic Examinations and Health-Related Characteristics in Patients Implanted with a Clear or Yellow Intra-Ocular Lens

	*Clear IOL*	*Yellow IOL*	p *value*
Model 1 Ophthalmic examinations			
Best corrected visual acuity (log MAR)			
Pre-operative better eye	0.10±0.35	0.18±0.26	<0.01^a^
Pre-operative worse eye	0.69±1.02	0.57±0.56	NS^a^
Post-operative better eye	−0.05±0.08	−0.03±0.08	NS^a^
Post-operative worse eye	0.12±0.68	0.10±0.30	NS^a^
Cataract type (%)			
Nuclear sclerosis (>Grade 2, Emery–Little classification)	38.7	15.6	<0.01^b^
Central cortical	45.2	48.9	NS^b^
Posterior sub-capsular	38.7	37.0	NS^b^
Model 2 health indices			
VFQ-25 total score			
Pre-operative	66.4±16.5	64.7±16.2	NS^a^
Post-operative			
2 months	80.4±12.8	80.2±12.0	NS^a^
7 months	80.6±14.8	81.5±12.6	NS^a^
PSQI global score			
Pre-operative	5.40±3.30	5.41±3.88	NS^a^
Post-operative			
2 months	4.30±3.43	5.38±3.70	NS^a^
7 months	4.80±2.84	5.27±4.27	NS^a^

Unless indicated otherwise, data are given as the mean±standard deviation (SD).

^a^ Unpaired *t*-test.

^b^ Chi-squared test.

IOL, intra-ocular lens; NS, not significant; MAR, minimal angle of resolution; BMI, body mass index; SBP, systolic blood pressure; VFQ-25, Visual Function Questionnaire 25; PSQI, Pittsburgh Sleep Quality Index; JDS, Japan Diabetes Society.

To investigate the potential effects of bilateral versus unilateral methodology on QOL, patients were classified into different groups (*i.e*., those undergoing their first surgery, those undergoing bilateral surgery, and those undergoing a second surgery) on the basis of the surgical plan for each patient. The “first surgery” group included patients undergoing their first and unilateral surgery. For patients to be classified as part of the “second surgery” group, there had to have been a washout period of >6 months after the first surgery. In the “bilateral” group, patients were undergoing surgery on their second eye 2–14 days after surgery on their first eye. Patients were required to complete the questionnaires before the first surgery and then again 2 and 7 months after the second surgery. To evaluate the contribution of seasonal differences on QOL, the seasonal distribution of surgeries was determined according to the date on which the surgery was performed; for this analysis, patients had completed the questionnaires 1 month prior to surgery. The study was done in the Tokyo area of Japan, latitude 35.68°N, where photoperiods vary from 4 to 6 hr of daylight over the year (the average from 1981 to 2010 reported by Japan Meteorological Agency).

Cataracts were classified as either posterior sub-capsular cataracts (PSC), nuclear sclerosis (Grade III or IV on the Emery Little classification), or a central cortical opacity disturbing the optical axis. Routine ophthalmic examinations were performed at each visit before and 2 and 7 months after surgery, and patients were asked to complete VFQ-25 and PSQI.

We calculated the change (Δ) in sub-scale scores by subtracting pre-operative values from post-operative values. As such, we were able to evaluate the effects of the surgery on QOL. Inter-individual changes cannot be compared directly because of differences in psychological and physical factors for each individual on each of the sub-scales.^1,2^

The surgical procedures for cataract surgery and IOL insertion consisted of phacoemulsification and aspiration, followed by IOL fixation of a clear UV-blocking lens (SA60AT; Alcon Laboratory, Fort Worth, TX) for patients at Mita Hospital or a yellow blue-light-blocking lens (SN60WF; Alcon Laboratory) for patients at Saitama Hospital. A 2.75-mm-width superior corneoscleral incision was made for procedures and IOL was fixated in the capsular bag in all cases. All procedures and examinations were performed by experienced ophthalmologists. Peri-operative medications were the same in all surgeries. The anesthetic used was topical 1% lidocaine. Post-operatively, 1.5% levofloxacin was used as an antibiotic for 1 month after surgery, and 0.1% diclofenac sodium anti-inflammatory eyedrops were used for 2 months after surgery.

Where appropriate, data are given as the mean±standard deviation (SD). Data were analyzed using analysis of variance (ANOVA), unpaired *t*-test, the chi-squared test, and the Kruskal–Wallis test. Correlations were evaluated using the Pearson product moment correlation test. All analyses were performed using StatFlex (Atech, Osaka, Japan) and SPSS version 21 (SPSS Inc., Chicago, IL), with *p*<0.05 considered significant.

## Results

**FIG. 1.**
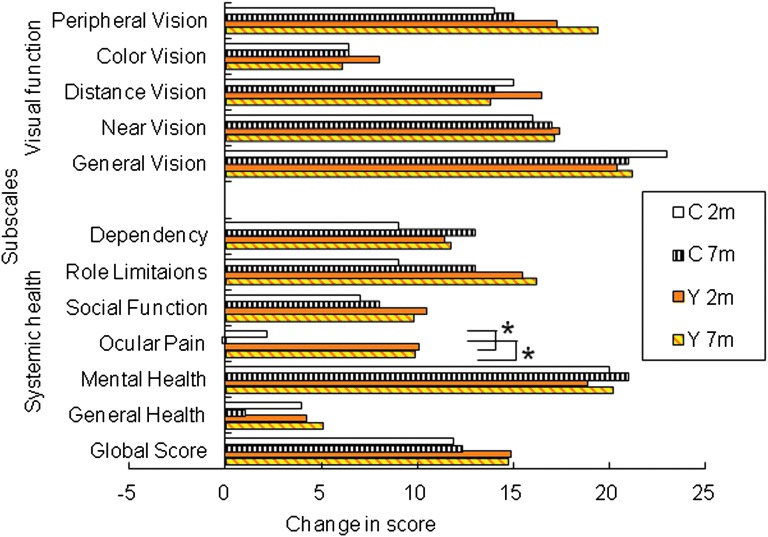
Changes in sub-scale scores on the National Eye Institute Visual Function Questionnaire (VFQ-25) after cataract surgery. Significantly greater improvements were seen in ocular pain scores 2 and 7 months after implantation of a yellow (Y) intra-ocular lens (IOL) than a clear (C) IOL (*p*<0.01 and *p*<0.05, respectively; unpaired *t*-test). There were no significant differences between the two groups for any of the other sub-scales. Data show mean differences in different sub-scale scores, calculated by subtracting post-operative values from pre-operative values, in patients implanted with a yellow IOL (*n*=135) or a clear IOL (*n*=71).

Analysis of sub-scales on the VFQ-25 revealed a significantly greater improvement in ocular pain scores 2 and 7 months after surgery in the case of patients implanted with a yellow IOL versus a clear IOL (*p*<0.01 and *p*<0.05, respectively; unpaired *t*-test; Fig. 1). The improvement in color vision was not significant in either group, and there were no significant differences between the two groups for any of the other sub-scales. There was >15 points improvement in scores for mental health, peripheral vision, distance vision, near vision, and general vision 7 months after surgery in both IOL groups (Fig. 1).

**FIG. 2.**
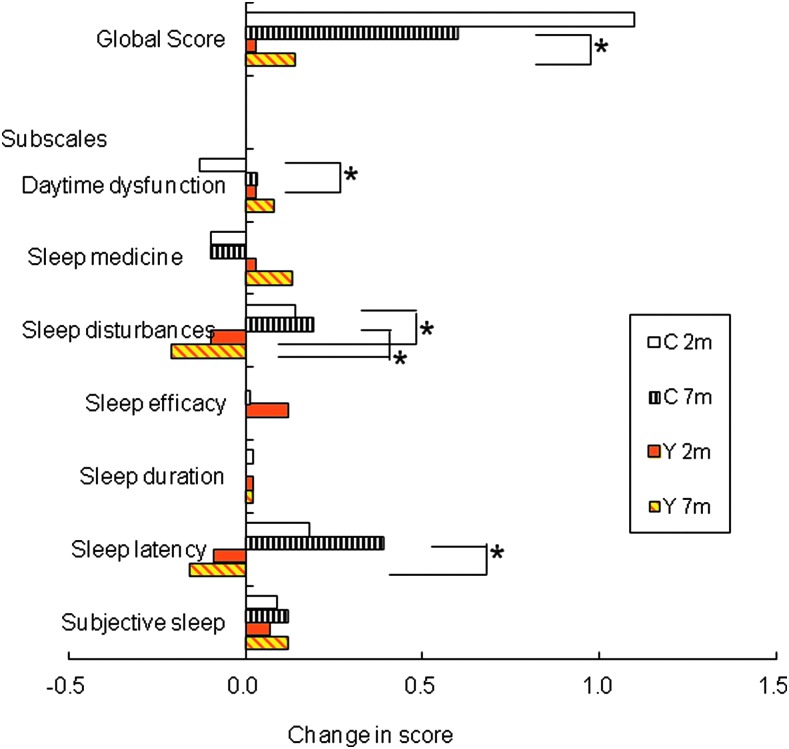
Changes in sub-scale scores on the Pittsburgh Sleep Quality Index (PSQI) after cataract surgery. Significantly greater improvements were seen for the sub-scale scores for sleep latency (*p*<0.001 at 7 months, unpaired *t*-test) and sleep disturbance (*p*<0.05 and *p*<0.01 at 2 and 7 months, respectively) in patients receiving a clear (C) intra-ocular lens (IOL) than those receiving a yellow (Y) IOL. Data show mean differences in different sub-scale scores, calculated by subtracting pre-operative values from post-operative values, in patients implanted with a yellow IOL (*n*=135) or a clear IOL (*n*=71).

On the PSQI, there were significantly greater improvements in the sub-scale scores for sleep latency (*p*<0.001 at 7 months, unpaired *t*-test) and sleep disturbance (*p*<0.05 and *p*<0.01 at 2 and 7 months, respectively), in patients implanted with a clear rather than yellow IOL (Table 2; Fig. 2). Daytime dysfunction improved in yellow IOL cases better than in clear IOL (*p*<0.05, unpaired *t*-test) 2 months after surgery.

**FIG. 3.**
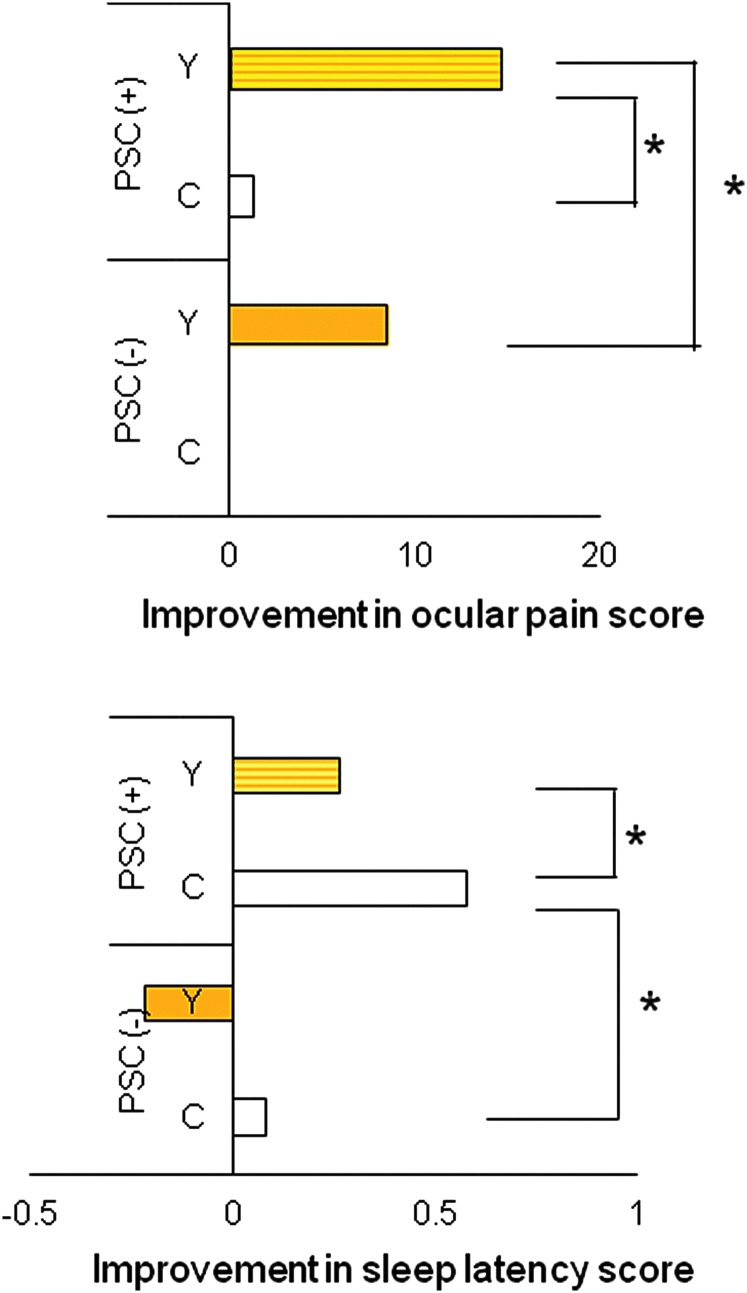
Improvements in ocular pain and sleep latency scores according to the presence of a posterior sub-capsular cataract (PSC) and the color of the intra-ocular lens (IOL) implanted. Greatest improvements in the ocular pain score were seen for patients with a pre-operative PSC who were implanted with yellow (Y) IOL. Sleep latency scores improved most markedly in patients with a pre-operative PSC who were implanted with a clear (C) IOL. Data show mean differences in sub-scale scores, calculated by the same formula as in Figs. 1 and 2, in patients implanted with a yellow IOL (*n*=135) or a clear IOL (*n*=71). (*) *p*<0.05 (unpaired *t*-test).

Stepwise multiple regression analyses of changes in health index sub-scales and related parameters revealed correlations between the change in sub-scale scores and the color of the IOL (Table 3). The change in ocular pain was correlated with age and the presence of a PSC. The change in sleep latency was correlated with the presence of a PSC. There were no significant correlations found for any of the other sub-scales. Figure 3 shows the improvement in ocular pain and sleep latency scores according to cataract type and IOL color. The greatest improvement in ocular pain scores was observed in patients with a PSC who received a yellow lens; in contrast, the greatest improvement in sleep latency scores was seen in patients with a PSC who were implanted with a clear IOL. The presence of a PSC was not correlated with the severity of these indices prior to surgery, but improvements after surgery were more prominent in patients with a PSC than in those without a PSC. To summarize, implantation of a yellow IOL favored improvements in ocular pain, the implantation of a clear IOL favored improvements in sleep latency and disturbances, and the presence of a PSC was a prognostic factor for ocular pain and sleep latency.

**Table 3.** Stepwise Multiple Regression Analysis of Changes in Sub-Scale Scores and Related Parameters

	*Δ Ocular pain score (VFQ-25)*		*Δ Sleep latency score (PSQI)*		*Δ Sleep disturbances score (PSQI)*	
	*β*	p *value*	*β*	p *value*	*β*	p *value*
Age	0.20	<0.01^*^	0.04	0.55	0.04	0.60
Sex^a^	−0.07	0.35	−0.04	0.60	0.16	<0.05^*^
Cataract type						
Nuclear sclerosis	0.05	0.56	−0.09	0.19	−0.10	0.22
PSC	0.03	<0.01^**^	−0.30	<0.01^**^	-0.10	0.92
Central cortical	0.16	0.06	−0.14	0.06	−0.01	0.93
IOL color^b^	0.22	<0.01^**^	0.15	<0.05^**^	0.15	<0.05^**^

Changes in health indices sub-scales were calculated by subtracting pre-operative values from values determined 2 months after surgery.

^a^ Male=1; female=0.

^b^ Yellow intra-ocular lens (IOL)=1; clear IOL=0.

^^*^,^**^^ *p*<0.05, Pearson product moment correlation test.

^^**^^ Adjusted for age and sex.

VFQ-25, visual function questionnaire 25; PSQI, Pittsburgh Sleep Quality Index; PSC, posterior sub-capsular cataract; IOL, intra-ocular lens.

**FIG. 4.**
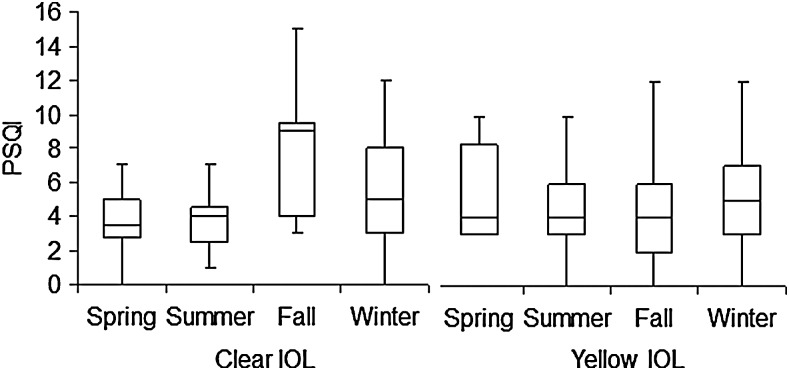
Box plot showing the seasonal distribution of pre-operative Pittsburgh Sleep Quality Index (PSQI) of patients undergoing cataract surgery for implantation of a clear or yellow intra-ocular lens (IOL). The horizontal line in each diagram indicates the median score on the PSQI. The height, positive error bar, and negative error bar of each box indicate the 25^th^–75^th^ percentiles, maximum value, and minimum value, respectively. Patients who had surgery in summer exhibited relatively better sleep quality than those undergoing surgery in winter, although the differences did not reach statistical significance.

There were no significant differences in the QOL of patients undergoing bilateral versus unilateral surgery (Table 4). Patients who had surgery in summer exhibited relatively better sleep quality than those who had surgery in the winter although the differences did not reach statistical significance (Fig. 4, Table 5).

**Table 4.** Quality of Life in Patients Undergoing Bilateral Versus Unilateral Surgery Following Implantation of a Clear or Yellow Intra-Ocular Lens

	*First surgery*	*Second surgery*	*Bilateral*	p *value*^a^
Clear IOL				
No. patients	37	9	25	
Male/female	18/19	3/6	9/16	
Age (years)	74.5±9.1	69.2±9.2	75.2±7.9	NS
VFQ-25 score				
Pre-operative	67.3±18.2	69.4±10.6	64.6±16.1	NS
Post-operative^b^	77.7±13.2	83.0±11.0	82.0±11.9	NS
Change^c^	10.4	13.6	17.4	
PSQI score				
Pre-operative	5.6±4.1	5.9±2.7	5.7±2.5	NS
Post-operative^b^	5.5±3.6	5.4±2.6	5.2±3.2	NS
Change^c^	−0.1	−0.5	−0.5	
Yellow IOL				
No. patients	31	19	85	
Male/female	13/18	5/14	35/50	
Age (years)	73.8±7.7	72.3±7.9	75.2±7.7	NS
VFQ-25 score				
Pre-operative	61.2±19.3	68.3±13.2	63.4±16.6	NS
Post-operative^b^	78.2±13.4	80.2±11.0	80.7±11.8	NS
Change^c^	17.0	11.9	17.3	
PSQI score				
Pre-operative	4.8±4.4	5.8±3.3	5.3±3.6	NS
Post-operative^b^	5.2±3.9	6.3±4.4	5.0±3.5	NS
Change^c^	0.4	0.5	−0.3	

Unless indicated otherwise, data are given as the mean±standard deviation (SD).

^a^ Kruskal–Wallis test.

^b^ Note, postoperative values are values determined 2 months after surgery.

^c^ The change in scores was calculated by subtracting preoperative values from postoperative values.

IOL, intra-ocular lens; NS, not significant; VFQ-25, Visual Function Questionnaire 25; PSQI, Pittsburgh Sleep Quality Index.

**Table 5.** Quality of Life in Patients Undergoing Surgery in Different Seasons and Implanted with a Clear or Yellow Intra-Ocular Lens

	*Season^a^ in which surgery was performed*				
	*Spring*	*Summer*	*Fall*	*Winter*	p *value^b^*
Clear IOL					
No. patients	18	13	15	25	
Male/female	9/9	8/5	3/12	10/15	
Age (years)	71.4±9.9	74.1±7.0	75.9±9.0	74.9±8.8	NS
VFQ-25 score					
Pre-operative	69.7±11.8	67.2±17.7	66.4±14.7	63.9±20.6	NS
Post-operative^c^	82.1±9.8	75.5±17.3	77.6±10.7	82.2±12.3	NS
Change^d^	12.4	8.3	11.2	18.3	
PSQI score					
Pre-operative	5.2±3.2	4.3±1.7	6.6±3.6	6.2±4.0	NS
Post-operative^c^	4.3±3.1	4.2±2.5	7.5±3.5	5.5±3.2	NS
Change^d^	−0.9	−0.1	0.9	−0.7	
Yellow IOL					
No. patients	9	56	45	25	
Male/demale	4/5	26/30	16/29	6/19	
Age (years)	74.4±9.1	74.4±8.6	75.1±6.8	73.9±7.1	NS
VFQ-25 score					
Pre-operative	53.7±19.3	63.9±17.5	65.6±16.4	63.2±14.6	NS
Post-operative^c^	80.3±15.1	78.3±12.7	82.7±9.6	79.3±13.3	NS
Change^d^	26.6	14.4	17.1	16.1	
PSQI score					
Pre-operative	6.9±5.0	4.9±3.3	5.3±4.1	5.5±3.4	NS
Post-operative^c^	5.4±5.4	5.1±3.0	5.3±4.0	5.1±4.1	NS
Change^d^	−1.5	0.2	0.0	−0.4	

Unless indicated otherwise, data are given as the mean±standard deviation (SD).

^a^ Spring, March–May; Summer, June–August; Fall, September–November; Winter, December–February.

^b^ Kruskal–Wallis test.

^c^ Note: Post-operative values are values determined 2 months after surgery.

^d^ The change in scores was calculated by subtracting pre-operative values from post-operative values.

IOL, intra-ocular lens; NS, not significant; VFQ-25, Visual Function Questionnaire 25; PSQI, Pittsburgh Sleep Quality Index.

## Discussion

The mean pre-operative PSQI (5.40 for clear IOL group and 5.41 for yellow IOL group) was around the cutoff value (5/6) in our study group, and we believe it is clinically significant that approximately half of the cataract patients had a sleep disorder given that the mean score of the normal control group in the PSQI validation study was 2.67.^25^ Thus, the prevalence of sleep disorder in cataract patients is relatively high, and it is of clinical interest to evaluate the pre-operative and post-operative sleep quality in these patients.

The results of the present study indicate differences in systemic health outcomes between clear and yellow IOLs. Implantation of a clear IOL had beneficial effects on sleep quality, whereas implantation of a yellow IOL had favorable effects in reducing ocular pain. These advantageous properties may also depend on fundus status, other local and systemic factors, and lifestyle. Although the differences may not directly lead to serious health problems, ophthalmic surgeons should be aware that altered light transmittance can have systemic effects after cataract surgery. According to previous reports,^26,27^ blue-light (400–500 nm) transmittance with a 20 diopter IOL is 21%–31% of solar energy compared with aphakic eyes for a yellow IOL and 42%–55% for a clear IOL.

It is paradoxical that the score of ocular pain improved after surgery in patients implanted with a yellow IOL. Post-operative eyes are inevitably painful with surgical wound, post-operative inflammation, and irritation by topical medications with preservatives.^28^ Our hypotheses for this paradox are mental improvement, response shift, and photophobia. Patients may feel relieved after surgery and simultaneously free from pre-operative stress. Patients' standard of response might change after surgery when they recognize how unconsciously they suffered from cataract-related painful symptoms including light sensitivity. With yellow IOL filters and more blue light than a clear IOL, some of the patients may answer with reduction of pre-operative glare or photophobia as a reduction of pain in VFQ-25.^29,30^

The greater improvement in sleep in patients implanted with a clear IOL may be due to better photoentrainment during the day and increased melatonin secretion before and during sleep. Our findings are supported by a large epidemiological study indicating daytime exposure to sunlight contributes to increased nocturnal melatonin secretion in older adults.^31^ The improvement in sleep was maintained in these patients for as long as 7 months. In contrast, improvements on the PSQI in patients implanted with a yellow IOL were not significant 7 months after surgery, and we speculate that the initial improvement in sleep was due mainly to patients' raised spirits.

Despite previous speculation that decreased light transmittance because of nuclear sclerosis may be the major factor underlying cataract-related sleep disorders,^32,33^ the presence of a PSC was significantly correlated with sleep latency in the present study, whereas nuclear sclerosis was not. Indeed, it is well known that PSC is the worst factor for poor scores on the VFQ-25^24^ and photophobia. Eye care personnel should be aware that removal of a PSC will predictably result in benefits to the patient.

In the present study, changes in sub-scale scores were analyzed because there were inter-individual differences for most scores, and a change in score was deemed to be a good indicator of surgical outcome. Moreover, because of the possibility of a “response shift,”^1,2^ there may have been changes in individual standards after the intervention. It was typical in cataract patients that the pre-operative ocular pain score was not correlated with the presence of a PSC, but patients with a PSC claimed greater improvement after surgery than those without a PSC. These effects were evidenced by the change in scores, rather than absolute post-operative scores. It is not uncommon that patients, prior to surgery, are unaware of the extent of their decreased vision and for them to be surprised at the improvement in their vision at their first post-operative examination. The greater impact of a PSC on QOL than nuclear sclerosis may be due to its rapid progression and detrimental effects on visual function and systemic health.

Bilateral versus unilateral surgery is a practical consideration when evaluating the systemic effects of cataract surgery.^34,35^ In the present study, we did not find any differences between patients undergoing bilateral versus unilateral surgery. However, it is interesting that pre-operative sleep quality seemed to exhibit seasonal differences. This could be explained by decreased light transmittance by the cataract^22,23^ in combination with short daylight hours exacerbating any sleep disturbances in cataract patients in winter.

The major limitations of the present study relate to the use of two separate study groups and the lack of randomization. The study was performed in two different medical centers, 20 km apart, in the same region. Although the surgeon, medical service, chronological factors, race, education, and insurance were exactly same for patients at both centers, there may have been differences in patient lifestyles and occupation. The sample sizes of the groups allocated a clear or yellow IOL were different (*n*=71 and *n*=135, respectively), but the groups were statistically comparable because the enrollment of consecutive surgical cases was not biased and appropriate statistical analyses were used. The present study was based on the results of questionnaires only. Restoration of photoreception in the eyes needs to be confirmed by a pupillometer, electroretinogram, optical coherence tomography, and/or other retinal and neurological examinations. Further investigation in large randomized control studies is necessary to confirm the different effects on systemic health of different-colored IOL.

In conclusion, the results of the present study indicate that the color of the IOL and cataract type are significantly correlated with non-visual prognosis. Ophthalmologists are encouraged to check for the presence of a PSC, photophobia, and sleep quality pre-operatively, because these factors could potentially be related to post-operative QOL after restoration of light transmittance in the eye.
